# Study on Effect of Striatal mGluR2/3 in Alleviating Motor Dysfunction in Rat PD Model Treated by Exercise Therapy

**DOI:** 10.3389/fnagi.2019.00255

**Published:** 2019-10-02

**Authors:** Ping Chen, Xiaodong Li

**Affiliations:** ^1^College of Sport Science, JiShou Univerity, JiShou, China; ^2^College of Physical Education and Sports, Beijing Normal University, Beijing, China

**Keywords:** 6-OHDA, exercise, rat Parkinson’s disease model, striatum, glutamate, mGluR2/3, motor dysfunction

## Abstract

**Background**: Exercise therapy has been widely applied in clinical rehabilitation as an important practical and side effect—free adjuvant therapy, with a significant effect in alleviating motor dysfunction of patients with Parkinson’s disease (PD) or animal PD models. This study focuses on the effect of exercise therapy in reducing the concentration of extracellular glutamate (Glu) in the striatum in a rat PD model by upregulating the expression of group II metabotropic Glu receptor (mGluR2/3), so as to alleviate motor dysfunction in the rat PD model.

**Methods**: Neurotoxin 6-hydroxydopamine (6-OHDA) was injected into the right medial forebrain bundle (MFB) of the rats to establish the semi-lateral cerebral damage PD model. The sham-operated group was given an equal amount of normal saline at the same site and taken as the control group. The apomorphine (APO)-induced rotational behavior test combined with immunohistochemical staining with tyrosine hydroxylase (TH) in the substantia nigra (SNc) and striatum was performed to assess the reliability of the model. The exercise group was given treadmill exercise intervention for 4 weeks (11 m/min, 30 min/day, 5 days/week) 1 week after the operation. The open field test (OFT) was performed to assess the locomotor activity of the rats; the Western blot technique was used to detect SNc TH and striatal mGluR2/3 protein expressions; real-time polymerase chain reaction (RT-PCR) was applied to detect striatal mGluR2 and mGluR3 mRNA expressions; the microdialysis—high-performance liquid chromatography (HPLC) method was adopted to detect the concentration of extracellular Glu in striatal neurons.

**Results**: Compared with the control group, the number of rotations of each model group at the first week was significantly increased (*P* < 0.01); compared with the PD group, the number of rotations of the PD + exercise group at the third week and the fifth week was significantly decreased (*P* < 0.05, *P* < 0.01). Compared with the control group, the total movement distance, the total movement time, and the mean velocity of each model group at the first week were significantly reduced (*P* < 0.05); compared with the PD group, the total movement distance, the total movement time, and the mean velocity of the PD + exercise group at the third week and the fifth week were significantly increased (*P* < 0.01). Compared with the control group, the count of immunopositive cells and protein expression of SNc TH, and the content of immunopositive fiber terminals in the striatal TH of each model group significantly declined (*P* < 0.01). Compared with the PD group, the striatal mGluR2/3 protein expression of the PD + exercise group significantly rose (*P* < 0.01). Compared with the control group, the concentration of extracellular Glu in striatal neurons of each model group at the first week significantly grew (*P* < 0.05); compared with the PD group, the concentration of extracellular Glu in striatal neurons of the PD + exercise group at the third week and the fifth week was significantly decreased (*P* < 0.01); compared with the PD + exercise group, the concentration of extracellular Glu in striatal neurons of the group injected with mGluR2/3 antagonist (RS)-1-amino-5-phosphonoindan-1-carboxylic acid (APICA) into the striatum at the third week and the fifth week was significantly increased (*P* < 0.05, *P* < 0.01). Compared with the control group, the striatal mGluR2/3 protein expression of the PD group was significantly downregulated (*P* < 0.01); compared with the PD group, the striatal mGluR2/3 protein expression of the PD + exercise group was significantly upregulated (*P* < 0.05); compared with the control group, the striatal mGluR3 mRNA expression of the PD group was significantly downregulated (*P* < 0.01); compared with the PD group, the striatal mGluR3 mRNA expression of the PD + exercise group was significantly upregulated (*P* < 0.01); 6-OHDA damage and exercise intervention had no significant effect on the striatal mGluR2 mRNA expression (*P* > 0.05). Compared with the PD + exercise group, the total movement distance, the total movement time, and the mean velocity of the PD + exercise + APICA group were significantly decreased (*P* < 0.05); compared with the PD group, the PD + exercise + APICA group had no significant change in the total movement distance, the total movement time, and the mean velocity (*P* > 0.05).

**Conclusion**: These data collectively demonstrate that the mGluR2/3-mediated glutamatergic transmission in the striatum is sensitive to dopamine (DA) depletion and may serve as a target of exercise intervention for mediating the therapeutic effect of exercise intervention in a rat model of PD.

## Introduction

Parkinson’s disease (PD) is one of the most common neurodegenerative diseases in the world and seriously affects the quality of life and the health of middle-aged and elderly people (Poewe et al., 2017; Haertner et al., 2018; Masilamoni and Smith, 2018; Oliveira de Carvalho et al., 2018; Stoessel et al., 2018). Currently, the pathogenesis of PD is still unclear (Carnwath et al., 2018), with a lack of an ideal therapeutic regimen in the clinic. According to most researchers, the primary pathologic changes of PD are that the degeneration and loss of dopaminergic neurons in the midbrain substantia nigra (SNc) cause the reduction of the dopamine (DA) release in the SNc—striatum pathway, the decrease of the direct pathway activity and the increase of the indirect pathway activity in the basal ganglia (BG), and the over-inhibition of thalamic and cortical neurons, which therefore lead to a clinical syndrome characterized by motor dysfunctions, such as bradykinesia, muscular rigidity, static tremor, gait disturbance, and postural instability (Ali and Morris, 2015; Hu et al., 2018; Stephano et al., 2018; Chen et al., 2019). Therefore, the main target for the treatment of PD is to deactivate the indirect pathway by enhancing dopaminergic neurotransmission or reducing glutamatergic neurotransmission.

Glutamate (Glu) is one of the primary excitatory neurotransmitters in the central nervous system. It plays a central role in the fundamental functions of the brain, including synaptic plasticity (critical for learning and memory), and the formation of neural networks during the development and repair of the central nervous system (McEntee and Crook, 1993; Meldrum, 2000). Due to the role of Glu in the neural circuits of the BG, it is also essential in motor control (Blandini et al., 1996). However, in some cases, over-continuous activation of Glu can damage nerve tissue and involve the occurrence of a variety of brain diseases (Blandini, 2010). According to the findings in recent years, in PD patients or neurotoxin-induced PD model animals, the depletion of SNc–striatum DA could cause the excessive activation of the cortex–striatum Glu pathway, release plenty of presynaptic Glu, and activate the striopallidal GABAergic pathway (the indirect pathway is overactive; Klockgether and Turski, 1989; Gerfen, 1992; Blandini et al., 2000; Wichmann and Delong, 2007). Therefore, blocking the excessive release of presynaptic Glu of the cortex–striatum pathway or inhibiting Glu’s effects could reduce glutamatergic transmission as well as indirect pathway activity. Glu exerts its biological effects through mediation of its receptors. Glu receptors are classified into iontropic Glu receptors (iGluRs) and metabotropic Glu receptors (mGluRs; Lau and Tymianski, 2010; Litim et al., 2017; Jenner and Caccia, 2019). In recent years, extensive studies have focused on the effect of iGluRs in the occurrence and development of PD and put forward that the excitotoxicity of Glu may be one of the important mechanisms in the occurrence and development of PD (DeLong and Wichmann, 2015; Van Laar et al., 2015). Many studies have indicated that although iGluR antagonist has an anti-PD effect, it is still restricted because the receptor is not specifically distributed in the central nervous system, and nonselective iGluR antagonist may have significant side effects, like cognitive dysfunction and psychotomimetic symptoms, in clinical experiments (Dell’anno et al., 2013; Masilamoni and Smith, 2018). Therefore, researchers have turned to mGluRs and found that mGluR2/3 may be an important target for the treatment of PD (Nicolletti et al., 2011). Group II mGluR is a cortex–striatum autoreceptor located at the presynaptic terminal, and its activation can reduce the cortex–striatum Glu release at the presynaptic terminal. Currently, mGluR2/3 agonist has been partially applied in clinical treatment, with a significant efficacy (Litim et al., 2017). According to the findings of an epidemiological survey, exercise/body movement can reduce the onset risk of PD (Lauzé et al., 2016); clinical and basic studies have verified that different forms of exercise/body movement can alleviate symptoms or delay the development of symptoms of patients with PD or animal PD models (Cheng et al., 2016; Sheibani et al., 2017). Therefore, it is inferred in this study that exercise intervention may have an effect in alleviating motor dysfunction in the rat PD model by upregulating the striatal mGluR2/3 protein expression, reducing the Glu release at the presynaptic terminal, and then decreasing the activity of the indirect pathway. In this study, *in vivo* microdialysis–high-performance liquid chromatography (HPLC), real-time polymerase chain reaction (RT-PCR), Western blot, and other molecular biological techniques were adopted to explore the effect of exercise intervention on the concentration of extracellular Glu in striatal neurons, the striatal mGluR2/3 mRNA expression, and the striatal mGluR2/3 protein expression in the rat PD model; the intervention with mGluR2/3 antagonist further confirmed the significant regulatory effect of mGluR2/3 on the concentration of extracellular Glu in striatal neurons and the motor function of the rats and provided experimental evidence for the hypothesis that exercise may alleviate the excitotoxicity caused by excessive activation of the cortex–striatum Glu at the synapse by upregulating mGluR2/3 and reducing the concentration of extracellular Glu in striatal neurons.

## Materials and Methods

### Experimental Animals

**Figure 1:**
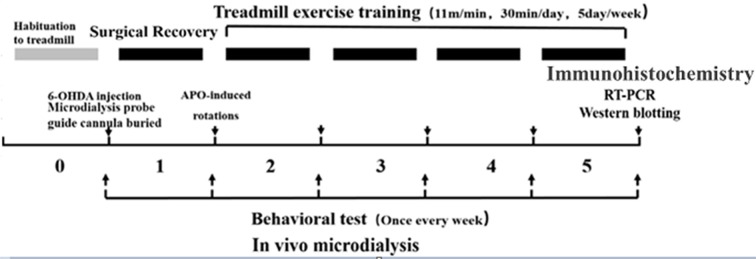
Experimental design flowchart.

Healthy clean-grade male SD rats weighing 240 ± 10 g (6 weeks old) were provided by Beijing HFK Bioscience Company Limited [Beijing, China; production license no. SCXK (BJ) 2009-0007]. The rats were fed in separate cages (three to four rats per cage), and kept on a 12:12 h light-dark cycle at a room temperature of 20–25°C with free access to food and water. During the experiment, the rats were given humanitarian care in the 3R principle for experimental animals. Before the formal experiment, they were enrolled in a 7-day adaptive exercise and forced treadmill exercise, and those incapable of finishing the preset treadmill exercise were excluded. The animal study was reviewed and approved by the experimental animal ethics committee, School of Physical Education and Sports, Beijing Normal University (IACUS-BNU-NKLCNL2016-02). The experimental design flowchart is as follows (Figure 1).

### Modeling and Assessment

The rats were fasted for 24 h with free access to water, and then intraperitoneally injected with 10% chloral hydrate (0.35 ml/100 g) for deep anesthesia, fixed in a prone position on a rat digital stereotaxic instrument (RWD, Shenzhen, China), and kept warm at 37°C with a thermostatic heating pad. At 30 min before injection with 6-hydroxydopamine (6-OHDA), they were intraperitoneally injected with desipramine (25 mg/kg) for protection of norepinephrine serotonergic neurons. Rat hair at the calvaria was shaved to expose the scalp; the skin of the operative area was disinfected with polyninylpyrrolidone; the scalp and periosteum were cut open with surgical scissors along the central line of the skull; and the bone surface was wiped with cotton balls dipped in hydrogen peroxide (H_2_O_2_) to fully expose the anterior and posterior fontanelles, and the anterior and posterior fontanelles were kept at the same horizontal level. Paxinos and Watson’s stereotaxic coordinates (Paxinos and Watson, 1997) of the right medial forebrain bundle (MFB) were anterior fontanel (AP): −4.3 mm, right (R): 1.5 mm, and deep (D): 7.6–7.8 mm. Based on the above coordinates, a skull hole was drilled by a dental drill and then injected with 4 μl 6-OHDA (2 μg/μl, including 0.02% ascorbic acid and 0.9% normal saline with an injection velocity of 0.5 μl/min). The sham-operated group was injected with 4 μl 0.9% normal saline (including 0.02% ascorbic acid) at the same site by the same method. After injection, the needle was retained for 10 min before slowly retreating (with the velocity at 1 mm/min), and then the skull hole was filled with biological silica gel.

**Figure 2:**
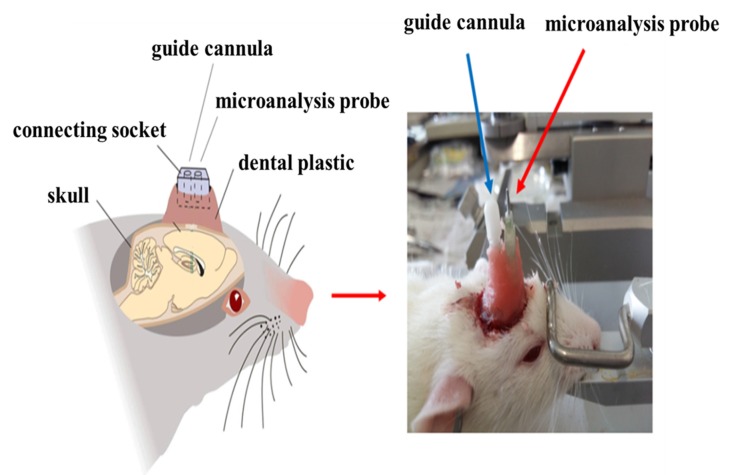
Imbedding of microdialysis cannula and drug delivery catheter.

After injection with 6-OHDA or normal saline, the microdialysis probe cannula and the mGluR2/3 antagonist [(RS)-1-amino-5-phosphonoindan-1-carboxylic acid, APICA] delivery catheter were implanted at the right striatum (AP: +1 mm, R: 2.5 mm, D: 3.5 mm; AP: −1.0 mm, R: +1.6 mm, D: 4.0 mm; Jia et al., 2017) and fixed at the skull surface by three to five small stainless steel screws and dental cement (Figure 2). After the operation, the rats were fed in separate cages and intraperitoneally injected with penicillin to prevent postoperative injection for successively 3 days.

At the seventh day after the operation, the apomorphine (APO)-induced rotational behavior test was performed to verify the successful establishment of the 6-OHDA semi-lateral cerebral damage model. The experiment was performed in a tranquil environment. APO solution (0.1 mg/100 g·wt·d) was injected subcutaneously into the neck of the rats, and the number of rotations (in which the rats rotated head to tail with the left posterior limb as the axis) for 30 min was recorded. In this study, the criterion for successful establishment of the rat PD model was the net number of rotations (namely, the number of counterclockwise rotations minus the number of clockwise turns rotations) >100 r/30 min.

### Grouping

The model rats were randomly divided into three groups: 6-OHDA sedentary group (PD, *n* = 12), 6-OHDA + exercise group (PD + Ex, *n* = 12), and 6-OHDA + exercise + mGluR2/3 antagonist group (PD + Ex + APICA, *n* = 12). The sham-operated group was taken as the sedentary control group (control, *n* = 12).

### Exercise Intervention and Dosage Regimen

At the first week after the operation, the exercise regimen designed by Tajiri et al. (2010) was adopted for treadmill exercise intervention in the PD + exercise group and the PD + exercise + APICA group. The exercise regimen lasted for 4 weeks (11 m/min, 30 min/day, 5 days/week, rest on Saturday and Sunday). The treadmill exercise intervention time was 16:00–18:00 in the afternoon of each exercise day. At 20 min before each exercise, the PD + exercise + APICA group was injected with mGluR2/3 antagonist APICA inside the striatum by a microinjection pump, with injection volume of 1 μl. The control group and the PD group were injected with the same volume of normal saline within the same period and put in the treadmill but in a sedentary state without treadmill exercise.

### Assessment of Rat Locomotor Activity

**Figure 3:**
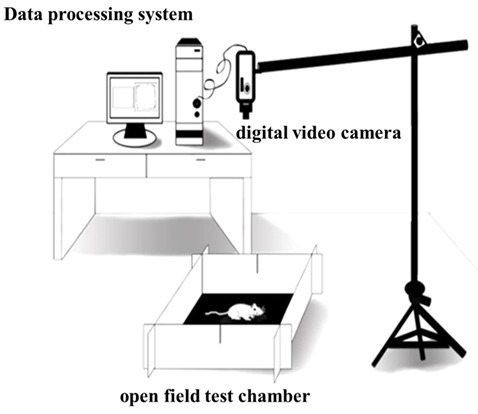
Rat locomotor activity detection mode.

The open field test (OFT) was performed to assess the locomotor activity of the rats (Sáenz et al., 2006). The OFT chamber (origin: Spain, brand: Panlab, supplier: RWD, Shenzhen, China) was 40 cm high and 100 cm wide and long, with gray non-transparent walls and a black bottom, and placed in a non-background anechoic chamber with a light intensity of 20 lux. A digital video camera [SONY (China) Company Limited, Shenzhen, China] was put at 80 cm above the OFT device and could cover the entire open field. After 60 min of the rats adapting to the experimental room, the rats were placed in the OFT chamber, and then after 0.5 min of being adapted to the open field, formal test recording was done. The Smart 3.0 software (origin: Spain; brand: Panlab; supplier: RWD, Shenzhen, China) was used to record the locomotor activity behaviors of the rats for 30 min, and the environment was kept tranquil during the whole test. After the test, built-in software Smart 3.0 was used to analyze the video of each rat (Figure 3).

### Striatal Microdialysis Sample Liquid Collection and Glu Concentration Determination

#### Microdialysis Cannula and Drug Delivery Catheter Imbedding Operation and Sample Collection

**Figure 4:**
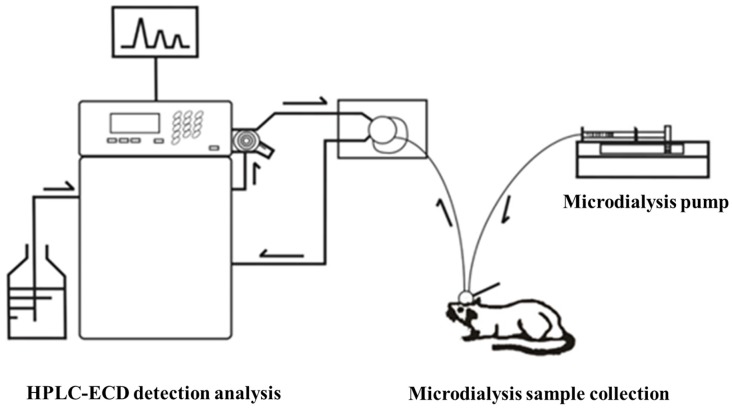
Microdialysis sample liquid collection of rat striatum in the waking state.

Before the collection of microdialysis samples, the guide probe core was removed, and then the probe was slowly inserted into the cannula and fixed. The rats were placed in the waking activity device. The probe input end was connected to the microinjection pump, and the output end was connected to the frozen collector. The microsyringe pump was filled with artificial cerebrospinal fluid, which was continuously perfused with a velocity of 0.2 μl/min. At 30 min after perfusion to achieve the balance state, the frozen collector began collecting microdialysis liquid samples with a velocity of 15 min per tube, one time per week, for four successive weeks. After collection, the samples were preserved at −80°C in a refrigerator (Figure 4).

### Rat Cerebral Histological Location

After the microdialysis sample collection, the rats were anesthetized with 10% chloral hydrate (0.35 ml/100 g); the thoracic cavity was cut open to expose the heart, which was perfused and fixed with 4% paraformaldehyde solution; and cerebral tissues were stripped and sunk in 30% sucrose-buffered paraformaldehyde solution overnight. The cerebral tissues were prepared into serial coronary frozen slices (40 μm), which were Nissl-stained to verify the locations of the microdialysis cannula and drug delivery catheter, and compared with cerebral stereotaxic coordinates, so as to eliminate the dialysate samples with microdialysis probe cannula and drug delivery catheter imbedding locations outside in the striatum.

### Glu Precolumn Derivatization Fluorescence Detection Method

**Figure 5:**
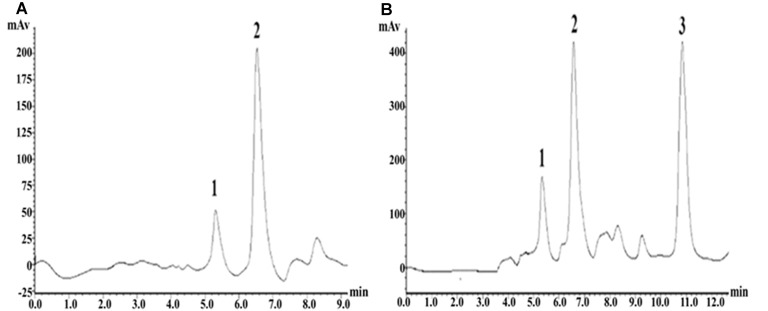
High-performance liquid chromatography (HPLC) fingerprints of Glu standard samples and striatal microdialysis liquid samples. Note: **(A)** HPLC fingerprints of glutamate (Glu) standard sample, in which 2 represents Glu. **(B)** Rat striatal microdialysis–HPLC, in which 2 represents Glu, and 1 and 3 are unknown.

The precolumn derivatization fluorescence detection method was used to determine the striatal Glu concentration. Glu’s chromatographic mobile phase was composed of 0.1 mol/L potassium dihydrogen phosphate solution (pH downregulated to 6.6) and pure methanol (40% for isorheic elution). Before use, it was filtered with 0.22 μm organic filter membranes and degassed through ultrasonic vibration. The flow rate was set at 1 ml/min. The SHIMADZU ODS-SP (4.6 × 150 mm, 5 μm) chromatographic column was adopted, with the column oven temperature at 25°C, the excitation wavelength at 357 nm and the emission wavelength at 455 nm. According to the precolumn derivatization method, 13.5 mg ortho-phthalaldehyde was first weighed and dissolved in 250 μl pure methanol; then, 25 ml prepared boric acid solution (0.4 mol/L) was added and mixed evenly; and finally, 100 μl β-mercaptoethanol was drop-wise added and protected from light at 4°C. Subsequently, 0.53 g Na_2_CO_3_ was weighed, fully dissolved in 100 ml ultrapure water, and prepared into Na_2_CO_3_ buffer (0.05 mol/L). Glu standard substances were accurately weighed and prepared into standard solutions (10, 1, 0.1, 0.01, 0.001 μmol/L), or the mother solution was first prepared and then diluted in sequence. Twenty microliters of dialysate/standard solution was weighed, added with 10 μl derivating agent and 10 μl Na_2_CO_3_ buffer (0.05 mol/L); they were fully mixed and put aside for 30 s; 20 μl was extracted for sampling. The concentration-peak area standard curve was drawn in LC-Solution software based on the corresponding peak areas of the five concentration standard samples. With the peak area as vertical coordinate Y and the standard sample concentration as horizontal ordinate X, the standard curve was *Y* = 1179939.70X − 19522.40 (*r*^2^ = 1.0000). The chromatographic peaks of the samples were qualitatively analyzed according to the Glu peak retention time; the area of the chromatographic peak with the same retention time (±0.1) as the standard sample was obtained by LC-Solution software, and the sample concentration was quantitatively based on the standard curve (Figure 5).

### TH and MGluR2/3 Detection

#### Immunohistochemistry

The immunohistochemical technique was adopted to detect the count of tyrosine hydroxylase (TH)—immunopositive cells of the SNc and the content of TH-immunopositive fiber terminals in the striatum. After the behavior test at the last week, the rats were fasted for 24 h, then intraperitoneally injected with 10% chloral hydrate (0.35 ml/100 g) for anesthesia, and perfused with 0.9% normal saline (250 ml) and 4°C 4% PFA solution (250 ml) *via* the ventriculus sinister through the ascending aortic cannulation. After the perfusion, the whole cerebral tissues were taken out and fixed in paraformaldehyde solution for 24 h. The cerebral tissues were dehydrated, trimmed and embedded after being taken out. When being sliced, the coronal plane of the cerebral tissues was first trimmed. Striatum and SNc locations were determined by reference to Paxinos and Watson’s (1997) stereotaxic coordinates. Serial coronary slices were made around each determined site, and one out of every other three slices was selected, with a thickness of 5 μm. The cerebral slices were rinsed with 0.01 M PBS (pH 7.4) and put in 0.3% Triton X-100 PBS at room temperature for 30 min for cell rupture. Then, they were incubated with 3% H_2_O_2_ and rinsed with PBS. The cerebral slices were transferred to PBS of 5% normal goat serum (haoranbio, China) for 1 h incubation at room temperature and then incubated with rat anti-TH monoclonal antibodies (1:3,000, Sigma, USA) overnight. After being rinsed with PBS three times, the cerebral slices were incubated with biotinylated rabbit antibodies (Millipore, USA) for 1 h at room temperature, then incubated with avidin-biotin-peroxidase compound (ABC-Elitekit, Vector Laboratories, Burlingame, CA, USA) for 1 h at room temperature, rinsed with PBS three times, and stained with DAB solution for 10–20 s. An Olympus-DP72 microscope (Olympus, Japan) was used for microphotography, and Image-Pro Plus 6.0 was adopted for statistics and analysis for the count of immunopositive cells of SNc TH and the content of immunopositive fiber terminals in the striatal TH (mean optical density), so as to determine the damage of dopaminergic neurons.

### Western Blot

Western blot technique was adopted to detect SNc TH and striatal mGluR2/3 protein expressions. In the last week, at 24 h after the behavior test, the rats were intraperitoneally injected with 10% chloral hydrate (0.35 ml/100 g) for deep anesthesia and decapitated; then, the cerebral tissues were taken out. The right striatum and the ventral mesencephalon were quickly stripped. After the protein content was determined by the bicinchoninic acid (BCA) method, the cerebral tissues were added with 5-fold SDS, boiled in water for 5 min, and then cooled and preserved in a refrigerator at −80°C. Thirty-microgram protein samples were taken for electrophoretic separation, placed on polyvinylidene fluoride (PVDF) membrane and then in plastic wrap, added with confining liquid, and slowly shaken at room temperature for 90 min. The membrane was sealed in 5% nonfat milk dissolved in Tris-buffered saline and Tween20 (TBST); added with rat primary antibodies TH (Abcam, UK) or rabbit primary antibodies mGluR2/3 (Millipore, USA), incubated at 4°C for 24 h, and rinsed with PBS; and then added with sheep anti-rat and sheep anti-rabbit secondary antibodies (Univ, China), placed on a shaker, incubated at room temperature for 90 min, and rinsed. After reaction at room temperature for 1 h, the membrane was added with chemical fluorescent liquid, followed by exposure and development with X-ray film. With β-actin as the internal control, ImageJ image analysis software was used to analyze the images. The relative protein content was represented by the ratio of the integral optical density (IOD) of each band to the IOD value of its corresponding β-actin.

### Real-Time Polymerase Chain Reaction

By reference to the literature of Zhang et al. (2009), SYBR Green I RT-PCR was used to detect the changes in the striatal mGluR2 and mGluR3 mRNA transcription levels. According to gene cDNA sequencing of *Rattus* listed on GenBank, Primer software was used to design primers. As for mGluR2, the upstream primer was 5′-TGGCACAGGCAAGGAGACAG-3′, the downstream primer was 5′-GCGATGAGGAGCACATTGTAGG-3′, and the amplified product size was 111 bp. As for mGluR3, the upstream primer was 5′-GAAGCCGAGTATATGTGTCCTG ATG-3′, the downstream primer was 5′-CACTGCTGTATGAACCACCAATGA-3′, and the amplified product size was 94 bp. As for the internal control GAPDH, the upstream primer was 5′-TGGAGTCTACTGGCGTCTT-3′, the downstream primer was 5′-TGTCATATTTCTCGTGG TTCA-3′, and the amplified product size was 138 bp.

### Statistics and Analysis

SPSS 20.0 software (SPSS Inc., Chicago, IL, USA) was adopted for statistics and analysis for all of the data. The results are represented by mean ± standard deviation ($\bar{x}$ ± SD). Sigmaplot 12.5 software was used for mapping. The means of groups were compared by one-way analysis of variance (ANOVA). Inter-group mean differences were compared by LSD test, while intra-group mean differences were compared by repeated-measure two-way ANOVA. The differences were considered statistically significant when the *P*-value was less than 0.05.

## Results

### Effect of Exercise Intervention in Alleviating Motor Dysfunction in Rat PD Model

**Figure 6:**
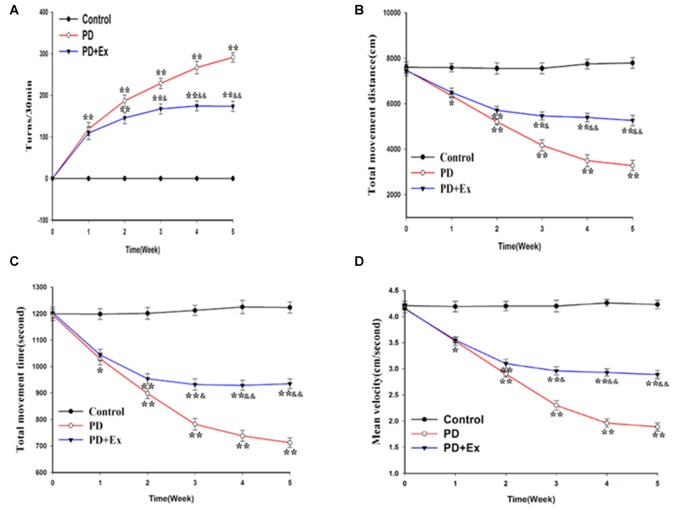
Effect of exercise intervention on motor dysfunction rat Parkinson’s disease (PD) model. **(A)** Apomorphine (APO)-induced rotational behavior of rats. **(B)** Changes in total movement distance of each group. **(C)** Changes in total movement time of each group. **(D)** Changes in mean velocity of each group. Compared with the control group, *P < 0.05 and **P < 0.01; compared with the PD group, ^&^*P* < 0.05 and ^&&^*P* < 0.01.

According to the results of the rotational behavior experiment, compared with the control group, the number of rotations of the PD group and the PD + exercise group at the first week was significantly increased, with statistically significant differences (*P* < 0.01); compared with the PD group, the number of rotations of the PD + exercise group at the third week was decreased, with statistically significant differences (*P* < 0.05); and the differences were very statistically significant at the fifth week (*P* < 0.01, Figure 6).

According to the results of the OFT, compared with the control group, the total movement distance, the total movement time, and the mean velocity of the PD group and the PD + exercise group at the first week were reduced, with statistically significant differences (*P* < 0.05); compared with the PD group, the total movement distance, the total movement time, and the mean velocity of the PD + exercise group were increased, with statistically significant differences (*P* < 0.05); and the differences were very statistically significant at the fifth week (*P* < 0.01, Figures 6B–D).

### No Effect of Exercise Intervention in Preventing Rat PD Model From Losing Dopaminergic Neurons

**Figure 7:**
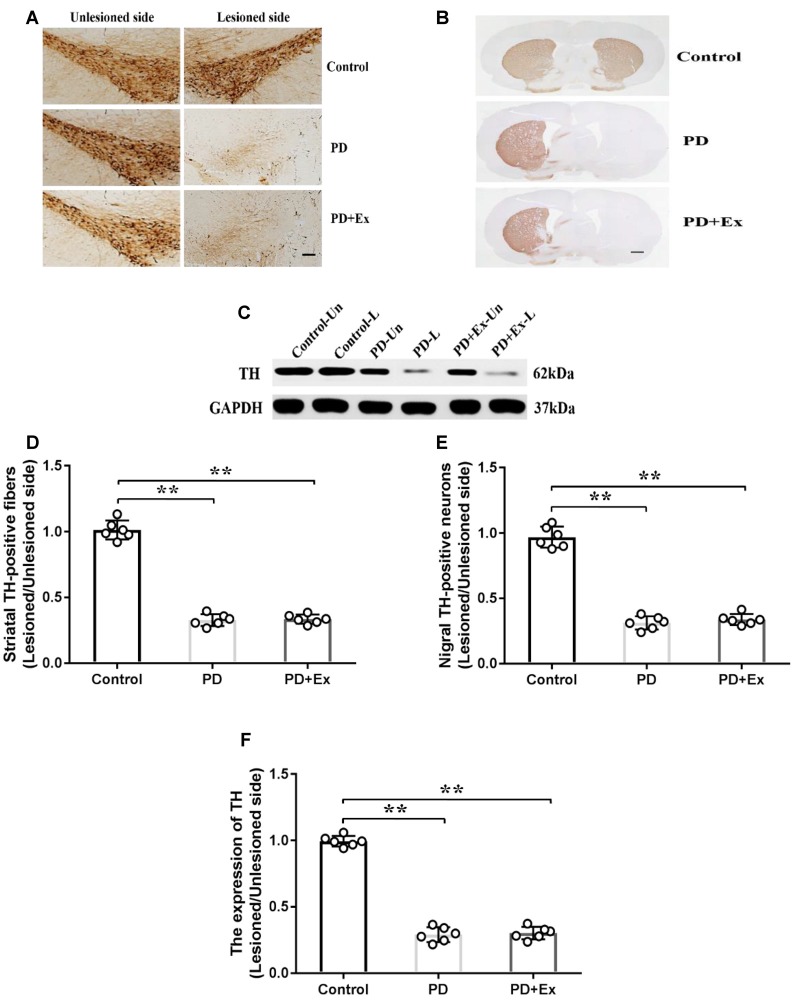
Effect of exercise intervention on substantia nigra (SNc)–striatum dopaminergic system in rat PD model. **(A)** Damaged side/undamaged side SNc tyrosine hydroxylase (TH)–immunopositive cell staining (scale bar = 50 μm). **(B)** Damaged side/undamaged side striatal TH-immunopositive fiber staining (scale bar = 50 μm). **(C)** Western blot of ventral mesencephalon TH protein expression. **(D)** Damaged side/undamaged side SNc TH-immunopositive cell optical density ratio. **(E)** Damaged side/undamaged side striatal TH-immunopositive fiber terminal content ratio. **(F)** Damaged side/undamaged side ventral mesencephalon TH protein expression ratio. Compared with the control group, **P < 0.01.

Compared with the control group, the count of immunopositive cells and protein expression of SNc TH, and the content of immunopositive fiber terminals in the striatal TH of the PD group declined, with statistically significant differences (*P* < 0.01); compared with the PD group, the count of immunopositive cells and protein expression of SNc TH, and the content of immunopositive fiber terminals in the striatal TH of the PD + exercise group had no significant change, with no statistically significant difference (*P* > 0.05, Figure 7).

### Effect of Exercise Intervention in Reducing Concentration of Extracellular Glu in Striatal Neurons in Rat PD Model

**Figure 8:**
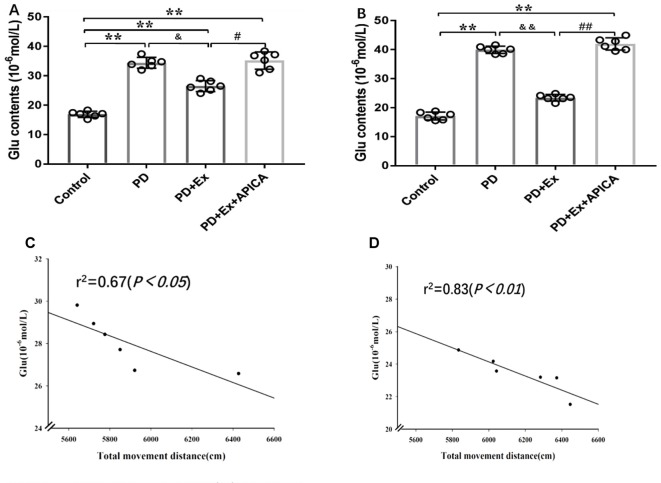
Effect of exercise intervention on concentration of extracellular Glu in striatal neurons in rat PD model. **(A)** Effect of 2-week exercise intervention on concentration of extracellular Glu in striatal neurons. **(B)** Effect of 4-week exercise intervention on concentration of extracellular Glu in striatal neurons. **(C)** Effect of 2-week exercise intervention on correlation between concentration of extracellular Glu in striatal neurons and locomotor activity in rat PD model. **(D)** Effect of 4-week exercise intervention on correlation between concentration of extracellular Glu in striatal neurons and locomotor activity in rat PD model. Compared with the control group, *P < 0.05; **P < 0.01; ^#^*P* < 0.05; ^##^*P* < 0.01; compared with the PD group, ^&^*P* < 0.05 and ^&&^*P* < 0.01.

Compared with the control group, the concentration of extracellular Glu in striatal neurons in the rat PD model at the third week and the fifth week significantly increased, with statistically significant differences (*P* < 0.01); compared with the PD group, the concentration of extracellular Glu in striatal neurons of the PD + exercise group at the third week and the fifth week significantly decreased, with statistically significant differences (*P* < 0.05, *P* < 0.01); compared with the PD + exercise group, the extracellular Glu in striatal neurons of the PD + exercise + APICA group at the third week and the fifth week was significantly increased, with statistically significant differences (*P* < 0.05, *P* < 0.01). Besides, the changes in the concentration of extracellular Glu in striatal neurons were negatively correlated with the changes in the locomotor activity of rats; with the increase of the exercise intervention time, the correlation was more significant at the fifth week (*P* < 0.05) than at the third week (*P* < 0.01, Figure 8).

### Effect of Exercise Intervention in Re-regulating Striatal mGluR2/3 Expression in Rat PD Model

**Figure 9:**
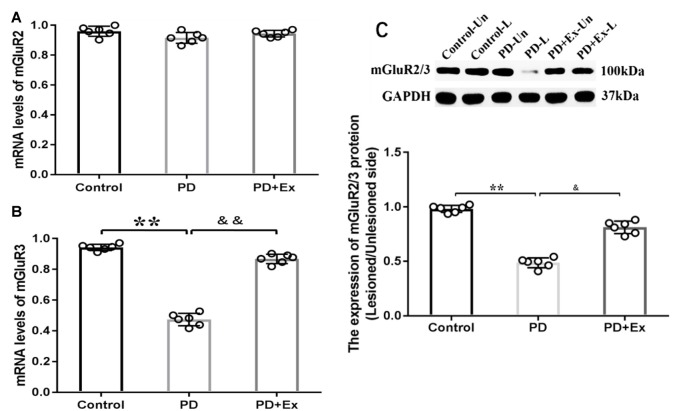
Effect of exercise intervention on striatal metabotropic Glu receptor (mGluR2/3) mRNA and protein expressions in rat PD model. **(A)** Effect of exercise intervention on striatal mGluR2 mRNA expression level in rat PD model. **(B)** Effect of exercise intervention on striatal mGluR3 mRNA expression level in rat PD model. **(C)** Effect of exercise intervention striatal mGluR2/3 protein expression level in rat PD model. Compared with the control group, **P < 0.01; compared with the PD group, ^&^*P* < 0.05 and ^&&^*P* < 0.01.

At the mRNA level, compared with the control group, the striatal mGluR3 mRNA expression level of the PD group significantly declined, with statistically significant differences (*P* < 0.01); compared with the PD group, the striatal mGluR3 mRNA expression level of the PD + exercise group was significantly increased, with statistically significant differences (*P* < 0.01). Both 6-OHDA damage and exercise intervention had no significant effect on the striatal mGluR2 mRNA expression level, with no statistically significant difference (*P* > 0.01). At the protein level, compared with the control group, the striatal mGluR2/3 protein expression of the PD group was decreased, with statistically significant differences (*P* < 0.01); compared with the PD group, the striatal mGluR2/3 protein expression of the PD + exercise group was increased, with statistically significant differences (*P* < 0.05, Figure 9).

### Effect of mGluR2/3 Antagonist in Preventing Exercise Intervention–Mediated Locomotor Activity Improvement in Rat PD Model

**Figure 10:**
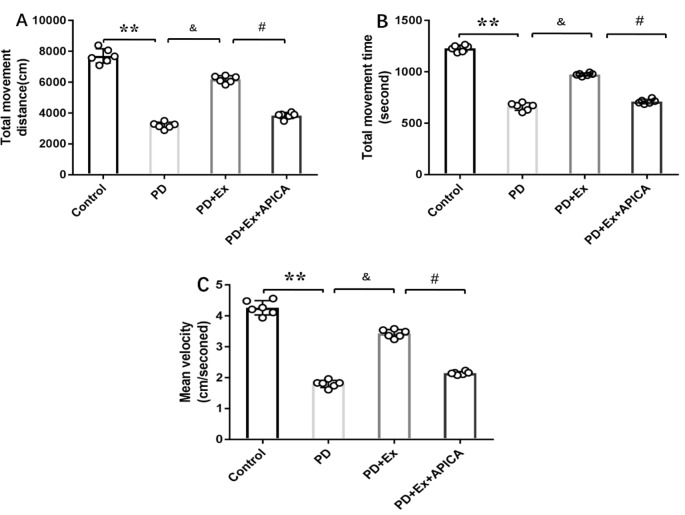
Effect of mGluR2/3 antagonist (RS)-1-amino-5-phosphonoindan-1-carboxylic acid (APICA) on locomotor activity of exercise-mediated rat PD model. **(A)** Effect of mGluR2/3 antagonist APICA on movement distance of exercise-mediated rat PD model. **(B)** Effect of mGluR2/3 antagonist APICA on movement time of exercise-mediated rat PD model. **(C)** Effect of mGluR2/3 antagonist APICA on mean velocity of exercise-mediated rat PD model. Compared with the control group, **P < 0.01; compared with the PD group, ^&^*P* < 0.05; compared with the PD + exercise group, ^#^*P* < 0.05.

Compared with the PD + exercise group, the total movement distance, the total movement time, and the mean velocity of the PD + exercise + APICA group declined, with statistically significant differences (*P* < 0.05); compared with the PD group, the PD + exercise + APICA group had no significant change in the total movement distance, the total movement time, and the mean velocity, with no statistically significant difference (*P* > 0.05, Figure 10).

### Effect of Exercise Intervention on Glu Release by Regulating mGluR2/3

**Figure 11:**
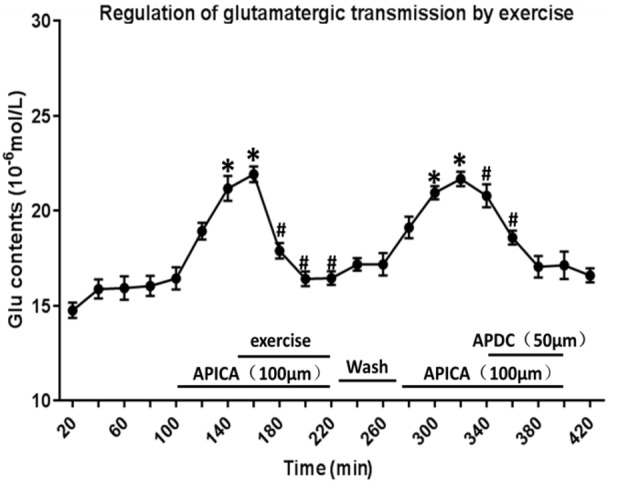
Effect of exercise on mGluR2/3 antagonist-induced extracellular Glu concentration in the striatum. mGluR2/3 antagonist APICA (100 μm) can increase extracellular Glu content in the striatum of normal rats. Exercise intervention or mGluR2/3 agonist aminopyrrolidine-2, 4-decarboxylate (APDC; 100 μm) can significantly reduce the increased concentration in the striatal extracellular Glu induced by mGluR2/3 antagonist (*n* = 6). Compared with the baseline, *P < 0.05; compared with APICA, ^#^*P* < 0.05.

Through reverse microdialysis, antagonist APICA was perfused to the striatum of normal rats to prevent mGluR2/3 and significantly increase the extracellular Glu content in the striatum. The treadmill exercise intervention could significantly reduce the extracellular Glu content in the striatum. After exercise, APICA was re-perfused to the striatum to increase the extracellular Glu content. However, the perfusion of mGluR2/3 agonist APDC could decrease the extracellular Glu content in the striatum (Figure 11).

## Discussion

This study explores the effect of the mGluR2/3-medicated glutamatergic system on dopaminergic neuron damage and exercise intervention’s efficacy in alleviating motor dysfunction in the rat PD model. According to the findings, exercise intervention alleviates motor dysfunction in the rat PD model, upregulates the striatal mGluR2/3 expression level, and reduces the concentration of extracellular Glu in striatal neurons. Besides, mGluR2/3 antagonist APICA prevents the effect of exercise intervention in alleviating motor dysfunction in the rat PD model. These findings verify that the effect of exercise intervention in alleviating motor dysfunction in the rat PD model is partially achieved by the mGluR2/3 dependency mechanism.

### Effect of Exercise Intervention in Alleviating Motor Dysfunction in Rat PD Model by Reducing Concentration of Extracellular Glu in Striatal Neurons

According to the findings of an epidemiological survey, people who are engaged in regular physical exercise in early age have a much lower incidence of PD than the general population (Sasco et al., 1992; Logroscino et al., 2006; Petzinger et al., 2015; Marica et al., 2018; Müller and Myers, 2018). Based on clinical observation, different forms of body movements (like treadmill exercise, resistive exercise, balance exercise, stretching exercise, deep breathing exercise, shadowboxing, dancing, and boxing) have a certain active effect in alleviating motor dysfunctions (such as bradykinesia, muscle rigidity, static tremor, titubation, and postural instability) of PD patients (Dashtipour et al., 2015; Hou et al., 2017; Giardini et al., 2018; Hirsch et al., 2018; Rawson et al., 2019). On the basis of the findings of animal PD model studies, different forms of exercise intervention have an active effect in alleviating motor dysfunction in animal PD models (Hsueh et al., 2018; Jang et al., 2018; Ohno et al., 2018; Speck et al., 2019). Yoon and Lee (2014) reported that 4-week balance and gait exercises can significantly alleviate motor dysfunction in an MPTP-induced rat PD model, which is manifested as a significant increase in the rat movement time in the OFT case and a significant reduction in the pole-climbing delay time; Viana et al. (2017) found that long-term moderate-intensity treadmill endurance exercise can significantly alleviate motor dysfunction in the mouse PD model, which is manifested as an increase in movement distance and time in the open field; Chuang et al. (2017) reported that 4-week treadmill exercise can significantly improve gait speed and balance in the 6-OHDA—induced rat damage model, which is manifested as a significant increase in the contact area of all left paws with the floor, the duration(s) of contact of the left paws with the floor, the swing speed of the left paws, and the distance between successive placements of the same paw, and a significant reduction in the distance between the two hind limbs and the average number of methamphetamine-induced rotations. According to the findings of this study, 4-week moderate-intensity treadmill exercise intervention can significantly alleviate motor dysfunction in the rat PD model, which is manifested as a significant increase in the movement distance, the movement time, and the movement velocity in the open field, and a significant decrease in the number of APO-induced rotations. A great number of clinical and basic studies have verified that many PD therapeutic regimens (drugs or surgeries) can alleviate motor dysfunction due to PD or progression of PD, but not all can improve the striatal DA concentration or the regeneration of dopaminergic neurons in the SNc compact part. Studies indicate that intracerebral injection of glial cell line-derived neurotrophic factor can relieve motor dysfunction due to PD but not increase the striatal DA level (Gash et al., 1996; Tseng et al., 1997). Likewise, deep brain stimulation also cannot increase the striatal DA level of PD patients (Sakellaridis, 2005; Chiken and Nambu, 2014). This study also found that exercise intervention has no significant effect on the loss of dopaminergic neurons and striatal dopaminergic nerve fibers in the SNc compact part of the rat PD model and cannot reverse the loss of striatal DA in the 6-OHDA-induced rat damage model. A lot of studies have indicated that exercise can enhance the DA use efficiency (Kim et al., 2014) or regulate presynaptic DA transporter and postsynaptic DA receptor (Rui et al., 2013), suggesting that exercise intervention’s efficacy in alleviating PD symptoms may involve other regulatory pathways or neurotransmitters more than the dopaminergic system. This study indicates that exercise intervention’s efficacy in relieving motor dysfunction due to PD may be correlated with the changes in the release of striatal Glu and acetyl choline (Sun et al., 2012). Therefore, exercise’s efficacy in alleviating motor dysfunction in the rat PD model is correlated with its effect on glutamatergic transmission and other systems rather than its direct effect on the loss of DA.

A study suggested that the loss of striatal dopaminergic neurons in the SNc causes the increase of striatal glutamatergic transmitters (Mao et al., 2013; Litim et al., 2017; Bagga et al., 2018). Specifically, the increase of cortex–striatum glutamatergic pathway activity and the higher extracellular Glu level in striatal neurons are correlated with the depletion of DA (Ossowska et al., 2002; Ambrosi et al., 2014; Melief et al., 2018; Jamwal and Kumar, 2019). On the basis of the findings of this study, the damage of dopaminergic neurons leads to the increase of extracellular Glu level in striatal neurons, which conforms to the findings of previous studies. This verifies that the damage of dopaminergic neurons can strengthen striatal glutamatergic transmission and involve cortex–striatum glutamatergic projection neurons in pathophysiology of PD. Therefore, the reversion of abnormal cortex–striatum glutamatergic transmission is regarded as one effective means to treat or alleviate PD. According to the findings of this study, exercise intervention can significantly reduce the extracellular Glu level in striatal neurons and improve the locomotor activity in rats. Furthermore, the locomotor activity in rats is negatively correlated with the extracellular Glu level in striatal neurons, which is more significant at the fourth week and the second week. This indicates that the inhibition of cortex–striatum glutamatergic transmission mediates the efficacy of exercise intervention in alleviating motor function in the rat PD model to some extent.

### Effect of GluR2/3 in Alleviating Motor Dysfunction in Rat PD Model

As the primary excitatory neurotransmitter in the central nervous system, Glu mainly exerts biological effects by combining with the corresponding receptors in the synaptic membrane. Glu receptors are classified into iGluRs and mGluRs (Lau and Tymianski, 2010; Lewerenz and Maher, 2015). In recent years, extensive studies have focused on the effect of iGluRs in the occurrence and development of PD and put forward that the excitotoxicity of Glu may be one of the important mechanisms in the occurrence and development of PD (Chotibut et al., 2014). Many studies have indicated that although iGluR antagonist has an anti-PD effect, it is still restricted because the receptor is not specifically distributed in the central nervous system, and nonselective iGluR antagonist may have significant side effects, like cognitive dysfunction and psychotomimetic symptoms, in clinical experiments (Du and Chen, 2017; Masilamoni and Smith, 2018). Therefore, researchers have turned to mGluRs and found that mGluR2/3 may be an important target for the treatment of PD (Chan et al., 2010). A study has suggested that mGluR2/3 is located on the presynaptic membrane as an autoreceptor for negative feedback control of Glu transmission, and its activation can reduce Glu release (Marino et al., 2002; Gasparini et al., 2013; Amalric, 2015). Currently, mGluR2/3 agonist has been partially applied in clinical treatment, with a significant efficacy (Samadi et al., 2009). A study showed that selective mGluR2/3 agonist reduced the overreaction of cortex–striatum nerve fibers after dopaminergic denervation (Conn et al., 2005; Litim et al., 2017). Senkowska and Ossowska (2003) reported that the activation of mGluRs in group II could relieve musculoskeletal rigidity and bradykinesia in the rodent PD model. Murray et al. (2014) found that injection of mGluR2/3 agonist in the lateral ventricles or SNc relieves reserpine-treated rats or 6-OHDA-induced rat damage models. Chang et al. (2006) reported that injection of mGluR2/3 agonist in the SNc could significantly reduce the forelimb application dissymmetry percentage and the net number of rotations toward the opposite side in the 6-OHDA rat model. According to the findings of this study, exercise intervention significantly increased the striatum mGluR3 mRNA expression level on the 6-OHDA-induced damage side and significantly upregulated the striatum mGluR2/3 protein expression on the damage side. Therefore, exercise intervention can strengthen the mGluR2/3 activity at local sites, and this is mainly achieved by affecting the striatal mGluR3 mRNA. It is inferred that the increased mGluR2/3 activity may automatically inhibit the presynaptic Glu release, so as to cause a lower extracellular Glu concentration and the changes in motor dysfunction. On the basis of the findings of this study, exercise intervention significantly reduced the concentration of extracellular Glu in striatal neurons in the 6-OHDA-induced rat damage model and significantly improved the locomotor activity of model rats. This study focused on exercise intervention in the rat PD model, as well as the administration of mGluR2/3 antagonist to the striatum at the 6-OHDA-induced damage side through the microinjection pump. Based on the findings, injection of mGluR2/3 antagonist in the striatum increased the concentration of extracellular Glu in striatal neurons and prevented the efficacy of exercise in alleviating motor dysfunction in the rat PD model. Therefore, mGluR2/3 participated in the exercise-mediated motor dysfunction alleviation in the rat PD model. This study provides direct evidence for the effect of mGluR2/3 in preventing the Glu release and the efficacy of exercise in preventing the cortex–striatum Glu release capability at the presynaptic terminal by increasing the local mGluR2/3 expression in the striatum.

## Conclusion

Exercise intervention can significantly alleviate motor dysfunction in the rat PD model, upregulate the striatal mGluR2/3 protein expression, and reduce the Glu concentration. mGluR2/3 antagonist can significantly increase extracellular Glu in striatal neurons and offset the beneficial effect of exercise in alleviating motor dysfunction in the rat PD model. Exercise intervention may exert an effect in alleviating motor dysfunction in the rat PD model by upregulating the striatal mGluR2/3 protein expression, reducing the Glu release at the presynaptic terminal, and relieving the excitotoxicity to the postsynaptic membrane.

## Data Availability Statement

The raw data supporting the conclusions of this manuscript will be made available by the authors, without undue reservation, to any qualified researcher.

## Ethics Statement

The animal study was reviewed and approved by Experimental animal ethics committee, school of physical education and sports, Beijing normal university.

## Author Contributions

PC: complete specific experiments and thesis writing. XL: the data processing.

## Conflict of Interest

The authors declare that the research was conducted in the absence of any commercial or financial relationships that could be construed as a potential conflict of interest.
